# Strategies for the synthesis of brevipolides

**DOI:** 10.3762/bjoc.17.157

**Published:** 2021-09-14

**Authors:** Yudhi Dwi Kurniawan, A'liyatur Rosyidah

**Affiliations:** 1 Research Center for Biomaterials, National Research and Innovation Agency, Cibinong, 16911, Indonesia; 2 Research Center for Biology, National Research and Innovation Agency, Cibinong, 16911, Indonesia

**Keywords:** brevipolides, 5,6-dihydro-α-pyrone, Furukawa-modified Simmons–Smith cyclopropanation, *Hyptis brevipes* Poit, ring-closing metathesis

## Abstract

In recent years fifteen 5,6-dihydro-α-pyrone derivatives, bearing either a distinctive cyclopropane or furan ring and named brevipolides A–O (**1**–**15**), have been isolated from the invasive plant *Hyptis brevipes* Poit. Their fascinating structural features, and the potent biological activities, including cytotoxicity against an array of human cancer cell lines and inhibition of the chemokine receptor CCR5, make them attractive synthetic targets. This review article highlights the recent synthetic methodologies and briefly summarizes their biological activities.

## Introduction

*Hyptis brevipes* Poit. is an invasive plant species belonging to the mint family *Lamiaceae* [1–4]. This plant has been reported to originate from tropical America but is now distributed broadly in other tropical areas of the world [1–5]. This herbaceous weed plant is often encountered in wastelands, plantation crops, orchards, forest verges, and grows abundantly in the fallow ground [1,3]. Hence, it is a potential threat to the crops and natural vegetation [1]. The extract of this suffruticose plant has long been used in folk medicines to treat headaches (Panama) and diarrhea (Paraguay) [6], for protection after giving birth (Panama, Indonesia, Malaysia) [6–9], against worms for newborn infants (Indonesia) [6], for prevention and treatment of different types of cancer (Indonesia) [10], asthma and malaria (southern Sahara) [2–3], and to combat intestinal parasites (Bolivia) [5]. In addition, this plant has also been used as a natural pesticide, particularly in cereal conservations, and to repel mosquitoes (southern Sahara) [2–3]. Biological activity investigations of the plant extract revealed various results, including toxicity to brine shrimp [9], DNA intercalation as well as antibacterial and fungal [4,9], and strong insecticidal activity against the 3rd instar larva of the cotton leaf worm *Spodoptera littoralis* (Biosd.) [7]. The essential oil extracted from the leaf of *Hyptis brevipes* Poit., furthermore, exhibited free radical scavenging and potential antitumor activities [2]. The ethnomedicinal background and preliminary biological studies triggered researchers to further examine the chemical constituents of the plant.

**Figure 1:**
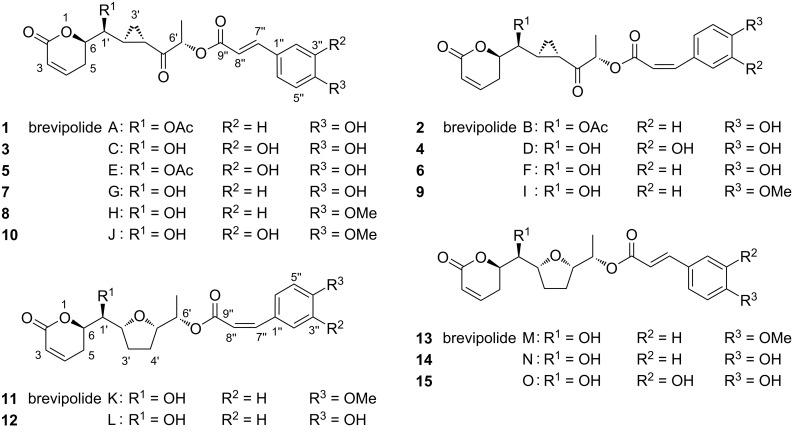
Structures of brevipolides A–O (**1** – **15**).

In 2009, Kinghorn and co-workers reported the first study to determine bioactive chemicals in *Hyptis brevipes* Poit. and isolated six new 5,6-dihydro-α-pyrone derivatives **1**–**6** along with other known compounds, including a 5,6-dihydro-α-pyrone derivative **7** [11], from the whole plant collected in Tawangmangu village, Indonesia (Figure 1) [4]. These six new compounds, **1**–**6**, were given the trivial names of brevipolides A–F, respectively, and the absolute configuration was determined by analysis of data obtained from their CD spectra and by Mosher’s ester formation, as C6*R*, C1’*S*, C2’*S*, and C4’*S*. The C6’ stereocenter at that time could not be established due to a rapid epimerization during cinnamate hydrolysis. Later, in 2013 Pereda-Miranda and co-workers isolated ten compounds, namely brevipolides A–J (**1**–**10**), from the aerial part of *Hyptis brevipes* Poit. collected in Mexico [12]. The C6’*S* configuration was then determined by X-ray crystallographic data of the hydrogenated brevipolide derivative. It is interesting to note that all the brevipolides A–J (**1**–**10**) pose the conserved stereocenters and bear a cyclopropyl unit in the core structure, which are in agreement with the prior structural assignment of compounds **7**–**9**, previously identified as unnamed inhibitors for the chemokine receptor 5 (CCR5), isolated from the Peruvian plant *Lippia alva* in 2004 [11]. Further, in 2017 Pereda-Miranda and co-workers isolated five more new 5,6-dihydro-α-pyrone derivatives, namely brevipolides K–O (**11**–**15**), from the same plant [1]. The structures have been determined by a comprehensive combination of quantum mechanical calculations and experimental spectroscopic analysis of their NMR and ECD data, to have a unique tetrahydrofuran ring instead of the cyclopropane functionality. The absolute configuration of these five compounds were evaluated and all conserved as C6*R*, C1’*S*, C2’*R*, C5’*S*, and C6’*S*.

Most brevipolide members exhibited cytotoxicity against various targets, including human colon, breast, laryngeal, cervix, prostate, and nasopharyngeal cancer cell lines with ED_50_ and IC_50_ values ranged in micromolar order [1,4,12]. One member showed activity in an enzyme-based ELISA NF-κB assay. Upon the mitochondrial transmembrane potential assay, three members demonstrated ED_50_ values in the nanomolar level [4]. Moreover, three of the members were identified as inhibitors of the chemokine receptor CCR5 [11]. Therefore, they are potential agents for treating human immunodeficiency virus (HIV).

The newly discovered brevipolides with interesting structural features in conjunction with promising biological activities have prompted the researchers to conduct synthetic studies. To date, there are six reported works in the literature for the syntheses of brevipolides, with the following details: two reports present the unsuccessful syntheses of brevipolide H [13–14] and four reports cover the successful syntheses of brevipolide H [15] and its enantiomer [16], brevipolide M [17], and brevipolide N [17–18], respectively.

To the best of our knowledge, this work presents the first review on the synthetic strategies to obtain brevipolides H, M, and N, aiming to achieve a deeper understanding of this area. It also provides a summary of the biological activities of brevipolides. This present review is chronologically organized, encompassing all synthetic works published since 2014.

## Review

### Syntheses

#### Kumaraswamy’s approach to brevipolide H (**8**)

**Scheme 1:**
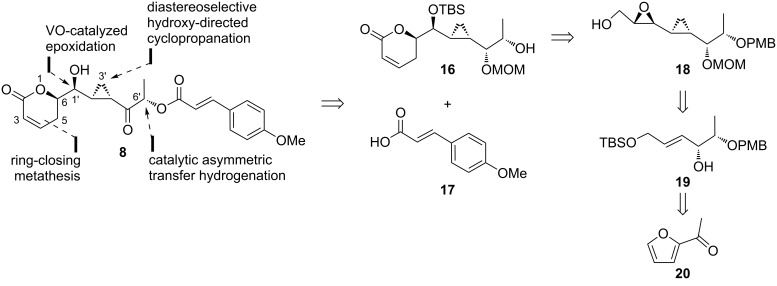
Retrosynthetic analysis of brevipolide H (**8**) by Kumaraswamy.

In 2014, Kumaraswamy and co-workers initiated the first attempt to synthesize brevipolide H (**8**, Scheme 1) [14]. In the retrosynthesis, the target compound **8** is achieved via esterification of the β-hydroxycyclopropyl intermediate **16** with the commercially available (*E*)-*p*-methoxycinnamic acid (**17**). The vital intermediate **16** is expected from cyclopropyl epoxy alcohol **18**, which in turn can be prepared from allylic alcohol **19** via the Furukawa-modified Simmons–Smith cyclopropanation and VO-mediated epoxidation. Acetylfuran (**20**) is chosen as the six-carbon precursor for the synthesis.

**Scheme 2:**
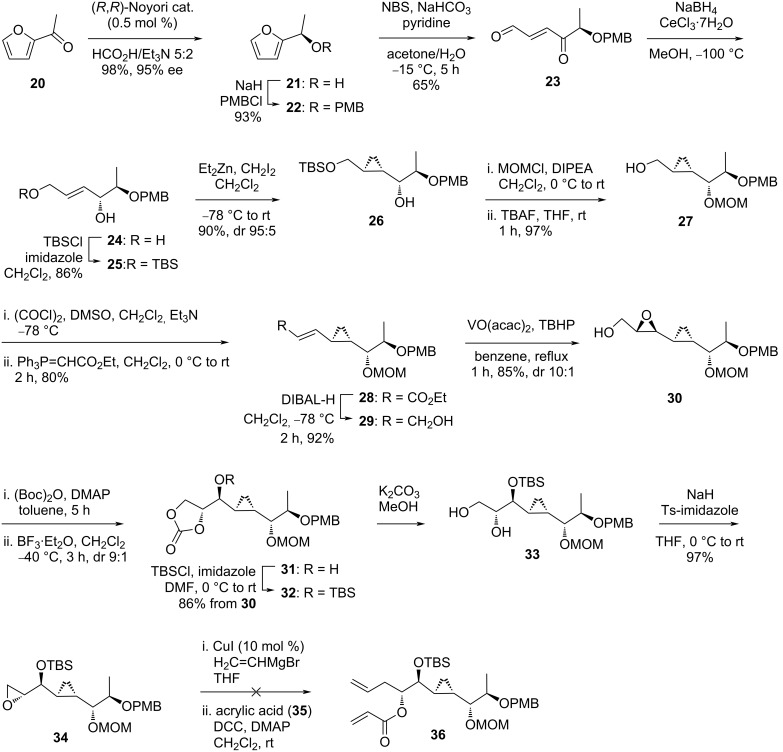
Attempt to synthesize brevipolide H (**8**) by Kumaraswamy. (*R*,*R*)-Noyori cat. = RuCl[*N*-(tosyl)-1,2-diphenylethylenediamine)(*p*-cymene)].

The forward synthesis transformed 2-acetylfuran (**20**) to its corresponding alcohol **21** through an asymmetric transfer hydrogenation catalyzed by a ruthenium complex (0.5 mol %) in 98% yield with 95% ee (Scheme 2). The azeotropic mixture of HCO_2_H/Et_3_N 5:2 was employed as the hydrogen source. Following protection of the alcohol moiety with PMBCl, ether **22** was realized in 93% yield. Afterwards, this species was transformed into the γ-keto α,β-unsaturated aldehyde **23** through an NBS-assisted furan oxidation procedure in moderate yield (65%). The keto functionality was reduced in a stereoselective manner adopting Luche conditions to provide diol **24** (dr 97:3), which after masking the primary alcohol moiety as a silyl ether, allowed isolation of the desired product **25** in 86% yield. This intermediate possessed the correct chirality on the free secondary alcohol to influence the stereo-outcome for the later cyclopropanation step. Thus, treatment of **25** with diethylzinc and diiodomethane delivered the expected *syn*-cyclopropyl carbinol **26** as the major diastereomer (dr 95:5) in 90% yield. After the protection of the secondary alcohol as MOM ether, the primary alcohol was liberated using TBAF to give compound **27** in 97% yield over two steps. The alcohol group in **27** was then oxidized to the corresponding aldehyde under Swern conditions and subsequently subjected to a Wittig reaction with a two-carbon phosphonium ylide reagent. The desired α,β-unsaturated ester **28** was then isolated in 80% yield over two steps. Reduction of the ester provided allylic alcohol **29** (92%) ready for later epoxidation. After considerable optimizations, the authors found that the dropwise addition of TBHP to **29** in refluxing benzene solution containing a catalytic amount of VO(acac)_2_ afforded the desired epoxide **30** after one hour in 85% yield (dr 10:1). This species was next converted to the terminal carbonate derivative **31** to transform the epoxy functionality to a vicinal diol through a two-step manipulation involving protection of the terminal alcohol as Boc derivative followed by BF_3_·Et_2_O-promoted intramolecular oxacyclization. After TBS protection, intermediate **32** was collected in 86% yield from epoxide **30**. Basic methanolysis of the cyclic carbonate followed by treatment with NaH and *N*-tosylimidazole then afforded terminal epoxide **34** in 97% yield. Unfortunately, attempts to open the epoxide using vinyl Grignard reagent followed by esterification with acrylic acid (**35**) proved to be inefficient due to low reproducibility and poor isolation of product **36**.

**Scheme 3:**
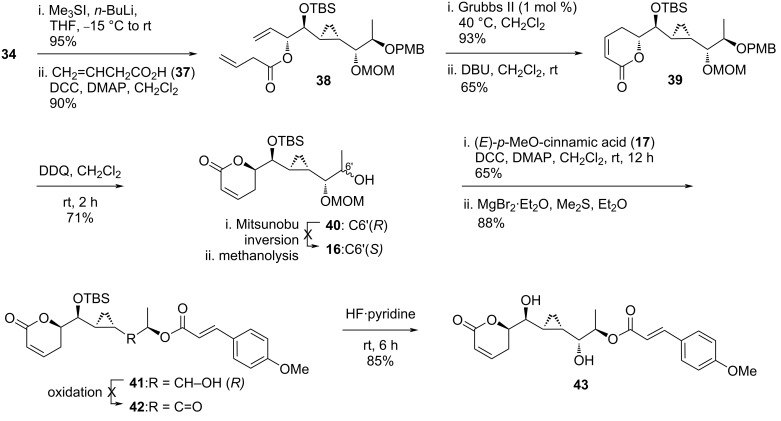
Attempt to synthesize brevipolide H (**8**) by Kumaraswamy (continued).

The strategy was altered by changing the terminal epoxide **34** to an allylic alcohol (95%) utilizing dimethyl sulfonium methylide followed by esterification with 3-butenoic acid (**37**) under Steglich conditions (Scheme 3). The resulting product **38** was isolated in 86% yield. A subsequent ring-closing metathesis reaction and DBU-assisted double bond migration then furnished the anticipated structure **39**. The PMB functionality was removed using DDQ to form alcohol **40**. At this point, the stereogenic center at the C6’ carbon required an inversion to match the target molecule. Thus, a standard Mitsunobu procedure followed by basic methanolysis were conducted. The desired inverted product **16**, however, did not form. The authors hypothesized a prospective possibility to obtain *ent*-**21** from precursor **20** by utilizing an antipode ligand in a Noyori reduction. As the continuation, intermediate **40** was coupled with (*E*)-*p*-methoxycinnamic acid (**17**) under Steglich conditions and treated with a Lewis acid to remove the MOM protection giving ester **41**. Oxidation of the secondary alcohol of this intermediate to its keto derivative was problematic and gave no desired product **42** after considerable experimentations. Eventually, the isolation of **43** marked the end of the synthetic study after treatment of **41** with HF·pyridine, which is the reduced form of 6’-*epi*-brevipolide H. Kumaraswamy and co-workers also performed a bioassay study for compounds **41** and **43** and found a higher cytotoxicity for the latter derivative against the MFC-7 cancer cell line.

#### Hou’s strategy to *ent*-brevipolide H (*ent*-**8**)

**Scheme 4:**
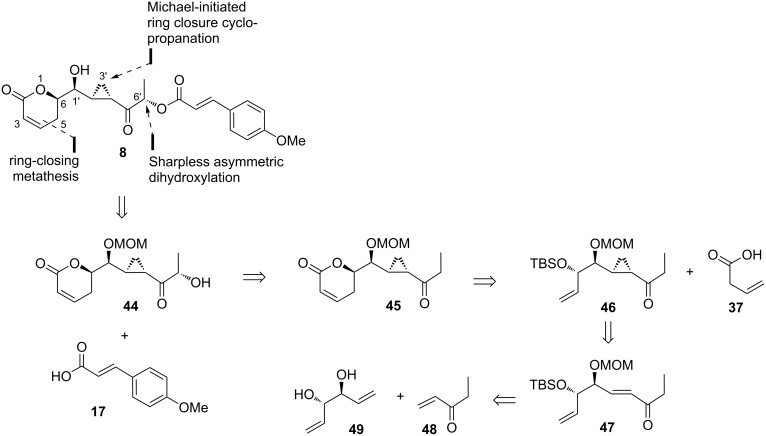
Retrosynthetic analysis of brevipolide H (**8**) by Hou.

Hou and co-workers, in 2014, demonstrated an efficient approach to synthesize brevipolide H (**8**), but unexpectedly ended up with the isolation of its enantiomer (*ent*-**8**) [16]. Common to the most of the reported retrosynthetic analyses of brevipolide, compound **8** is disconnected at the cinnamate ester bond giving β-hydroxy cyclopropyl intermediate **44** and (*E*)-*p*-methoxycinnamic acid (**17**) (Scheme 4). The β-hydroxy moiety in **44** can be installed via Sharpless dihydroxylation of the silyl enol ether derived from ketone **45**. The 5,6-dihydro-α-pyrone group in ketone **45** is envisaged from protected diol **46** by the sequence of Mitsunobu esterification, ring-closing metathesis, and base-promoted double bond migration. The cyclopropyl functionality in **46** can be assembled from the reaction of sulfur ylide and the α,β-unsaturated ketone **47**, which in turn can be realized from the cross metathesis between commercially available ethyl vinyl ketone (**48**) and the *C*_2_-symmetrical diene-diol **49**.

**Scheme 5:**
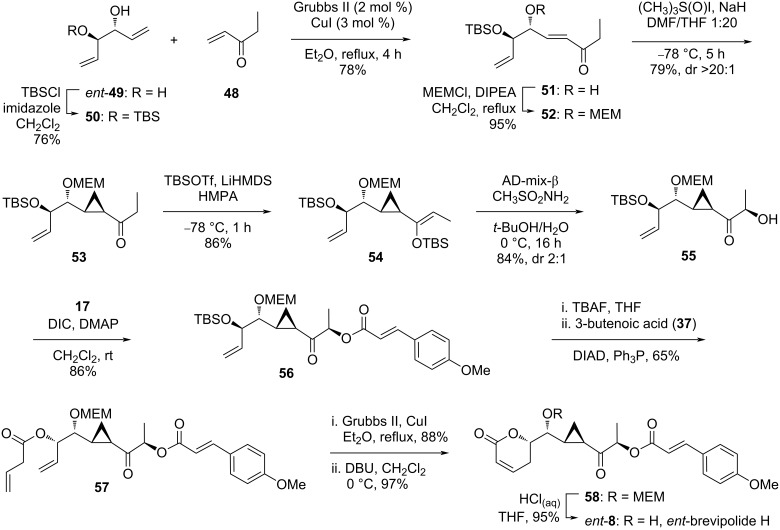
Synthesis *ent*-brevipolide H (*ent*-**8**) by Hou.

The synthesis commenced with the monoprotection of the known diene-diol *ent*-**49** as TBS ether affording compound **50** in 76% yield, which served as an excellent strategy to direct the later cross metathesis reaction to one site of the olefin over another (Scheme 5). Thus, the desired α,β-unsaturated ester **51** was afforded in 78% yield as an (*E*)-isomer exclusively after reaction with excess ethyl vinyl ketone (**48**) in the presence of a catalytic amount of Grubbs II catalyst and CuI. The free secondary alcohol in **51** required a protection prior to the next transformation. For this purpose, MEMCl was chosen instead of the previously arranged MOMCl, as the MEM functionality was found to have a better impact on the stereoselectivity of the later cyclopropanation reaction. Thus, the MEM ether **52** was obtained in 95% yield and subjected to in situ prepared dimethyl sulfoxonium methylide at low temperature to affect the Michael-initiated ring closure cyclopropanation at the more electrophilic olefin. The α-keto cyclopropyl intermediate **53** was formed in 79% yield with a dr value of >20:1. Reducing the proportion of DMF to 5% with respect to THF in the reaction mixture was hypothesized as the key factor for maximizing the *anti*-addition of the sulfoxonium ylide to **52**. Hou highlighted that the good diastereoselectivity control for the sulfoxonium ylide addition to acyclic α,β-unsaturated substrates such as **52** observed in their work represented the first example in literature. Hereupon, deprotonation of **53** over LiHMDS followed by addition of TBSOTf at low temperature successfully formed the (*Z*)-silyl enol ether **54**. Application of the Sharpless asymmetric dihydroxylation, promoted by AD-mix-β, gave the expected β-(*R*)-hydroxy cyclopropyl product **55** in 84% yield with moderate diastereoselectivity (dr = 2). The formation of ester **56** was achieved through reaction of **55** with the pre-activated acid **17** with *N*,*N’*-diisopropylcarbodiimide (DIC) and DMAP. Removal of the TBS protection with a fluoride source and succeeding Mitsunobu inversion with 3-butenoic acid (**37**) went smoothly to give ester **57**. This intermediate accommodated all the centers of the stereochemical brevipolides but in the mirror image of the natural configuration. After sequential ring-closing metathesis, base-promoted olefin migration, and MEM removal, the *ent*-brevipolide H (*ent*-**8**) was obtained. This compound showed inhibition activity in the preliminary assay against the cell proliferation of the human hormone-refractory prostate cancer cell line (PC-3).

#### Mohapatra’s strategy to brevipolide H (**8**)

**Scheme 6:**
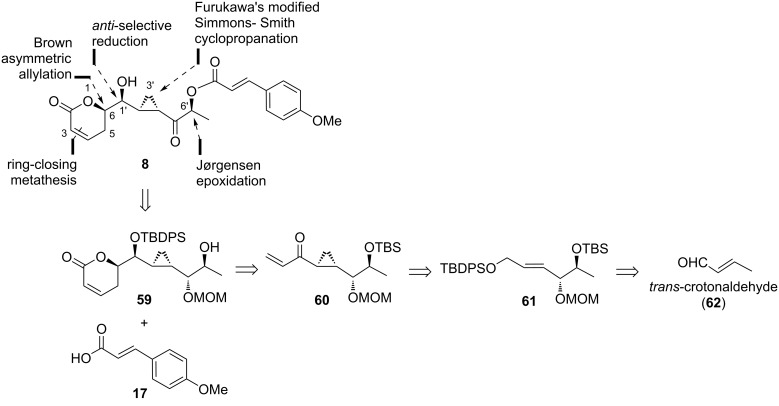
Retrosynthetic analysis of brevipolide H (**8**) by Mohapatra.

As part of the interest in synthesizing biologically active cyclopropane-containing natural products, Mohapatra and co-workers in 2015 started their attempt to prepare brevipolide H (**8**) [13]. The retrosynthesis started with envisaging a common 12-carbon intermediate **59** which can be derived from unsaturated ketone **60** (Scheme 6). The cyclopropyl moiety in **60** can be realized from allylic alcohol **61** via the Furukawa’s modified Simmons–Smith cyclopropanation protocol. Eventually, *trans*-crotonaldehyde (**62**) is selected as the precursor for this study.

**Scheme 7:**
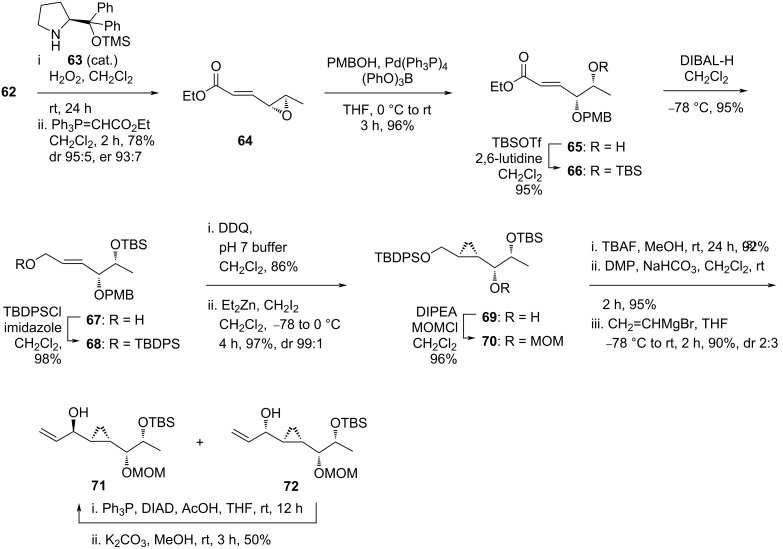
Attempt to synthesize brevipolide H (**8**) by Mohapatra.

The study began with the enantioselective epoxidation of *trans*-crotonaldehyde (**62**) under Jørgensen conditions using organocatalyst **63**, followed by a two-carbon homologation to obtain α,β-unsaturated epoxy ester **64** in 78% yield over two steps (dr 95:5, de 93:7) (Scheme 7). Then, the epoxide ring was opened regioselectively by *p*-methoxybenzyl alcohol utilizing a palladium(0) catalyst to afford the secondary allylic alcohol **65** in 96% yield. The free alcohol group was protected as *tert*-butylsilyl (TBS) ether **66** (95%) and the ester group was reduced to the primary alcohol **67** (95%). After protection as *tert*-butyldiphenylsilyl (TBDPS) ether **68** (98%), a Simmons–Smith cyclopropanation was attempted yet no desired product was obtained. Hence, the PMB-protecting group was first removed and the cyclopropyl product **69** was successfully attained in 97% yield with very high diastereoselectivity (dr 99:1). The free secondary alcohol group was re-protected as MOM ether **70** in 96% yield. After removal of the TBDPS group, the resulting free primary alcohol was oxidized under Dess–Martin conditions followed by Grignard reaction with vinylmagnesium bromide. The allylic alcohol products **71** and **72** were obtained as a diastereomeric mixture in 86% yield with poor stereoselectivity.

**Scheme 8:**
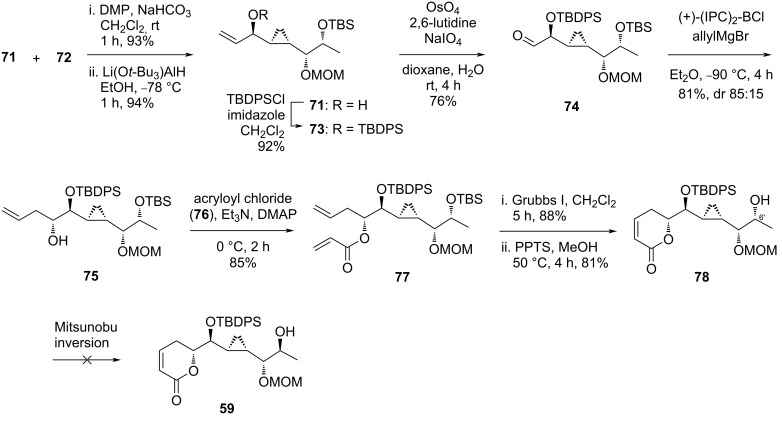
Attempt to synthesize brevipolide H (**8**) by Mohapatra (continued). (+)-(IPC)_2_-BCl = (+)-*B*-chloro-diisopinocampheylborane.

To gain more of the desired diastereomer **71**, the mixture of **71** and **72** was subjected to a two-step procedure involving a Dess–Martin oxidation followed by stereoselective reduction (Scheme 8). Among a selection of reagents, Mohapatra found that lithium tri-*tert*-butoxyaluminum hydride in ethanol at low temperature furnished **71** as a single diastereoisomer in 94% yield. The allylic alcohol moiety was protected as TBDPS ether **73** (92%) and oxidatively cleaved following Jin’s one step dihydroxylation–oxidation protocol using a NaIO_4_/(cat.) OsO_4_ system. Allylation of the resulting aldehyde **74** was best performed under Brown’s protocol at low temperature utilizing a chiral allyl reagent prepared from allylmagnesium bromide and (+)-*B*-chloro-diisopinocampheylborane. By this route, the alcohol product **75** was isolated in 81% yield (dr 85:15). Esterification of this molecule with acryloyl chloride (**76**) went smoothly providing the diene **77** (85%), which, after the sequential ring-closing metathesis with Grubbs I catalyst and TBS removal, gave the 5,6-dihydro-α-pyrone **78** in 71% yield over two steps. The stereocenter at the C6’ carbon demanded an inversion to fit the natural form. The standard Mitsunobu inversion protocol, unfortunately, failed to reach the expected transformation. The isolation of **78** ended their attempt to synthesize brevipolide H.

#### Hou’s strategy to brevipolide H (**8**)

**Scheme 9:**
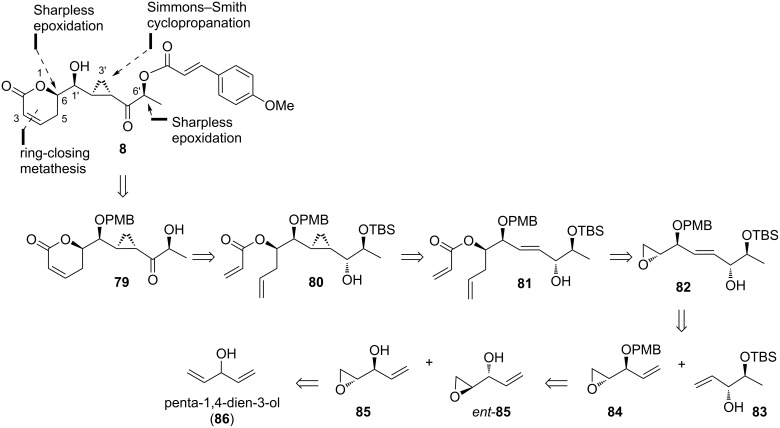
Retrosynthetic analysis of brevipolide H (**8**) by Hou.

Two years after the successful synthesis of *ent*-brevipolide H (*ent*-**8**), in 2016, Hou and co-workers reported the first total synthesis of natural brevipolide H (**8**) [15]. The retrosynthesis was initiated by disconnection of the cinnamate ester bond to give intermediate **79**. The 5,6-dihydro-α-pyrone moiety is obtained via ring-closing metathesis reaction of acrylate ester **80**, which in turn can be proposed from allylic alcohol **81** via Furukawa’s modified Simmons–Smith cyclopropanation (Scheme 9). The species **81** can be constructed through the sequential epoxide opening and esterification of compound **82**. This molecule is expected to be available from cross metathesis of olefins **83** and **84**. These two intermediates can then be readily prepared from optically active oxirane **85** and its enantiomer which can be derived through the Sharpless epoxidation of penta-1,4-dien-3-ol (**86**).

**Scheme 10:**
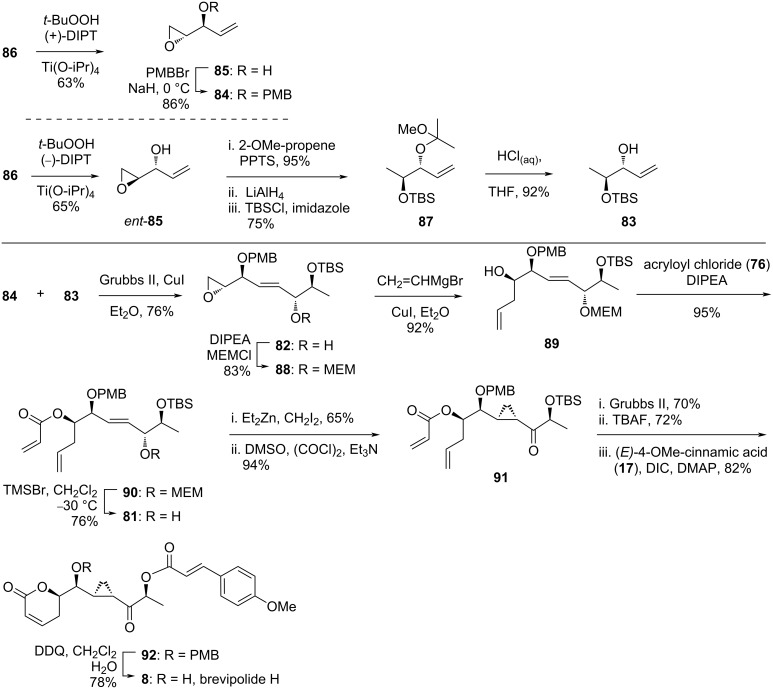
Synthesis of brevipolide H (**8**) by Hou.

In the synthesis, desymmetrization of **86** was achieved under Sharpless epoxidation conditions employing a *t*-BuOOH/(+)-DIPT/Ti(O-iPr)_4_ system to give epoxide **85** (63%), which was subsequently protected as PMB ether **84** in 86% yield (Scheme 10). In parallel, the same precursor **86** was subjected to another Sharpless epoxidation using (−)-DIPT affording *ent*-**85** in 65% yield. A series of functional group transformations involving hydroxy group protection, reduction of the epoxide, protection of the resultant free alcohol as TBS ether, and removal of the acetal protection afforded the expected allylic alcohol **83**. Accordingly, cross-metathesis reaction between **83** and **84** was successfully achieved utilizing Grubbs II catalyst to give the adduct **82** in 76% yield. The *E*/*Z* ratio for this compound was determined from the ^1^H NMR spectral analysis as >20:1. After protection of the secondary alcohol as MEM ether **88** (83%), the epoxide ring was opened with vinylmagnesium bromide to give the allylic alcohol **89** (92%). The acrylate ester **90** was smoothly obtained from this molecule in 95% yield and deprotected using TMSBr to give the allylic alcohol **81**. Subjection of this compound to a system containing Et_2_Zn/CH_2_I_2_ successfully furnished the expected *syn* adduct in 65% yield as a single diastereomer. Oxidation of this molecule under Swern conditions proceeded smoothly giving ketone **91** in 94% yield, which was highlighted as an important observation as the same transformation using the similar compound was reported to be unsuccessful [14]. Ring-closing metathesis of this compound installed the 5,6-dihydro-α-pyrone moiety, and TBS removal followed by esterification with 4-methoxycinnamic acid provided compound **92**. After removal of the PMB group using DDQ, the natural brevipolide H (**8**) was successfully achieved.

#### Sabitha’s strategy to brevipolide M (**13**)

**Scheme 11:**
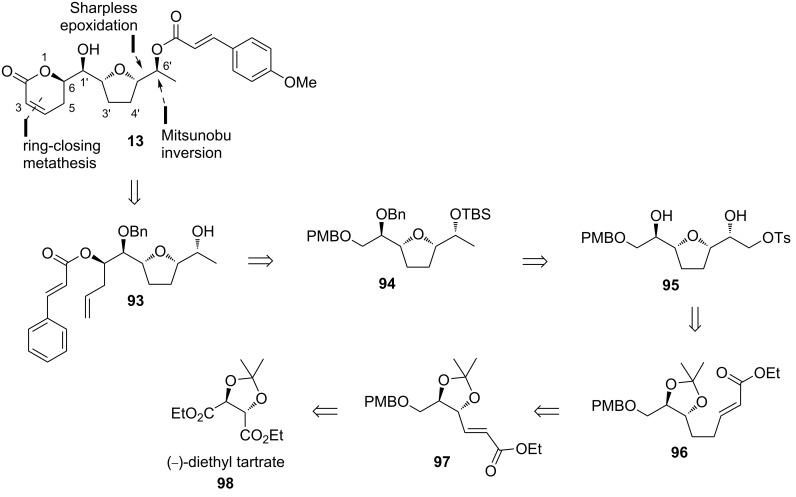
Retrosynthetic analysis of brevipolide M (**13**) by Sabitha.

Sabitha and Raju demonstrated the first total synthesis of brevipolide M (**13**) in 2017 [17]. In the retrosynthesis, compound **93** is hypothesized from the Mitsunobu inversion at the C6’ stereocenter with (*E*)-*p*-methoxycinnamic acid (**17**) and ring-closing metathesis of tetrahydrofuran **93** (Scheme 11). The olefin moieties can be installed via stereoselective allylation and cinnamic acid esterification of **94**, which is derived from symmetrical alcohol **95**. The right-hand side portion of this molecule can be constructed by epoxidation of the allylic alcohol derived from α,β-unsaturated ester **96** and the cyclization to form the furan ring is then expected to occur concurrently. (−)-Diethyl tartrate (**98**) was eventually selected as the precursor to construct the unsaturated ester **96** after a double two-carbon homologation.

**Scheme 12:**
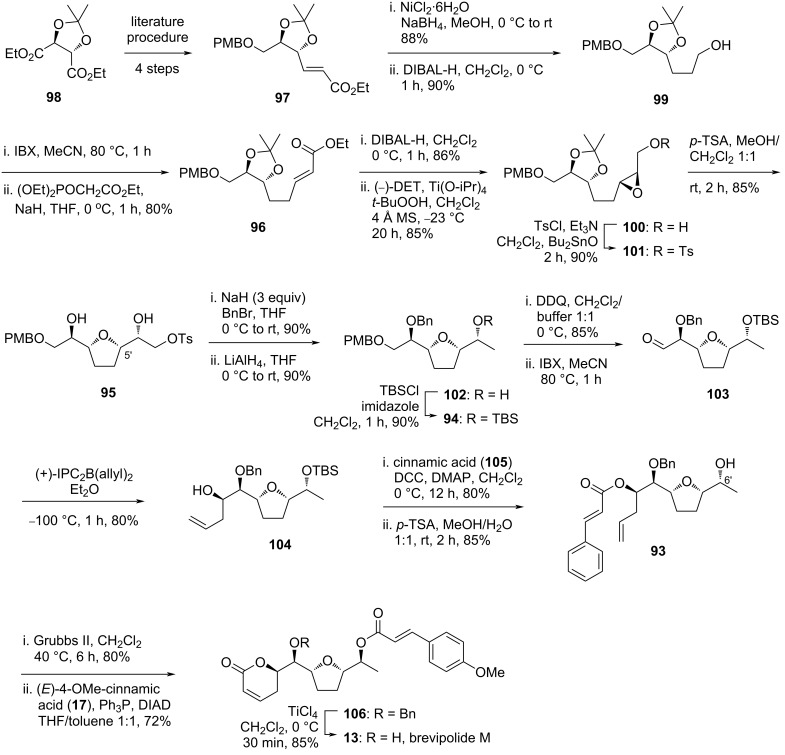
Synthesis of brevipolide M (**13**) by Sabitha.

In the reverse order, the synthesis of **13** began with the conversion of diethyl tartrate **98** into the PMB-protected α,β-unsaturated ester **97** in four steps adopting the literature procedure (Scheme 12). The chemoselective reduction of the olefin in **97** was furnished in 88% yield applying NiCl_4_·6H_2_O/NaBH_4_ as reagents in methanol followed by reduction of the ester part with DIBAL-H providing the primary alcohol **99** (90%). Oxidation of this moiety with IBX to its corresponding aldehyde served as a substrate for the two-carbon homologation via Wittig reaction giving ester **96** in 80% yield over the two steps. After reduction of the ester group to its primary alcohol counterpart, the Sharpless epoxidation protocol was applied to the double bond using (−)-DET to afford chiral oxirane **100** in 85% yield. The free primary alcohol was then protected as tosyl ester **101** and treated with *p*-TSA to induce intramolecular cyclization. The anticipated furan **95** was successfully isolated in 85% accompanied with the desired stereochemistry inversion at the C5’ carbon. Treatment with excess NaH furnished a terminal epoxide derivative which after the sequential treatment with benzyl bromide and reduction with LiAlH_4_ afforded alcohol **102** (81% yield from **95**). The free secondary alcohol was then protected as TBS ether **94** (90%) and the PMB ether was cleaved to liberate the primary alcohol. After being oxidized with IBX, the aldehyde **103** was isolated in 68% yield over two steps. Application of the asymmetric Brown’s allylation afforded **104** in 80% yield (dr 95:5) that was readily esterified to its cinnamate ester derivative in 80% yield. The TBS protecting group was removed under acidic conditions to give secondary alcohol **93** (85%). Afterwards, the 5,6-dihydro-α-pyrone functionality was constructed by applying a cross-metathesis protocol and the stereochemistry at C6’ was inverted with (*E*)-*p*-methoxycinnamic acid (**17**) via a Mitsunobu esterification. The resulting product **106** contained all the correct stereochemistry, which after removal of the benzyl protection, provided the target molecule brevipolide M (**13**) as a colorless oil.

#### Sabitha’s strategy to brevipolide M and N (**13, 14**)

**Scheme 13:**
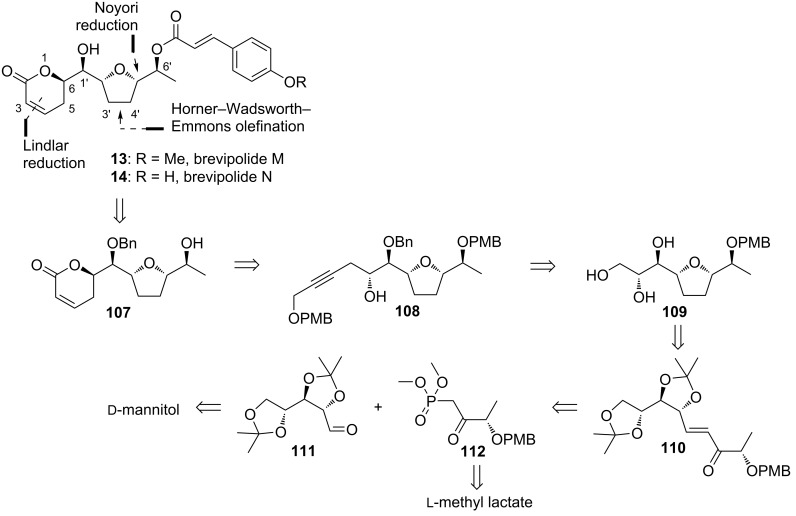
Retrosynthetic analysis of brevipolides M (**13**) and N (**14**) by Sabitha.

Following the previous success, Sabitha and Raju reported another approach to synthesize brevipolide M (**13**) which was shorter and more efficient than the former strategy [18]. Furthermore, this improved strategy was applied to achieve the first total synthesis of brevipolide N (**14**) by utilizing a different acid counterpart in the late esterification step. In the retrosynthesis, compound **107** is conceived as the branching intermediate to access both brevipolides M and N (Scheme 13). The 5,6-dihydro-α-pyrone moiety can be derived from alkyne **108** via the sequential deprotection, Lindlar reduction, and oxidation. The propargylic alcohol moiety is introduced by the addition of a protected propargyl alcohol to the epoxide derived from triol **109**. The furan ring is formed by an acid-catalyzed intramolecular cyclization of the alcohol intermediate obtained from the Noyori reduction of α,β-unsaturated ketone **110**. This compound is eventually constructed through Horner–Wardsworth–Emmons olefination of the known precursors **111** and **112**, which are prepared from ᴅ-mannitol and ʟ-lactic acid methyl ester, respectively.

**Scheme 14:**
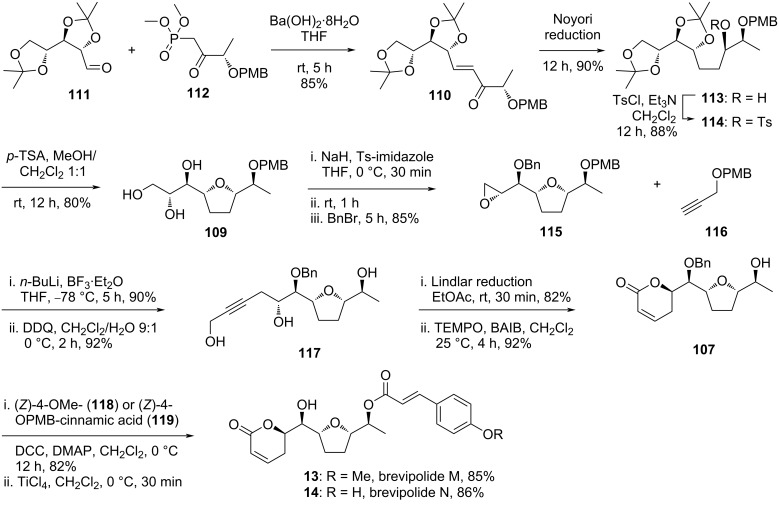
Synthesis of brevipolides M (**13**) and N (**14**) by Sabitha.

The synthesis commenced with the reaction between aldehyde **111** and phosphonate ester **112** using Ba(OH)_2_·8H_2_O to provide the unsaturated ketone **110** in 85% yield (Scheme 14). Application of a tandem 1,4/1,2-reduction to this compound under Noyori conditions gave the expected diastereomer **113** as the major product in 90% yield (dr 96:4). After protection of the resulting free secondary alcohol as the tosyl ester **114** (88%), this molecule was treated with an acid to remove the isopropylidene protection and induce intramolecular cyclization to the 2’,5’-*syn*-furan **109** in 80% yield. This triol was subjected to excess sodium hydride and tosylating agent, and the mixture was allowed to react for 90 minutes, after which benzyl bromide was added to furnish the terminal epoxide **115** in 85% from **109**. Then, the epoxide ring was opened with deprotonated propargylic ether **116**. Global removal of the PMB functionality with DDQ gave triol **117**. The partial reduction of the triple bond in **117** to the (*Z*)-olefin derivative was achieved using Lindlar catalyst and subsequent oxidation of the primary alcohol with the TEMPO/BAIB system facilitated the formation of pyrone **107**. Initially, Sabitha and Raju intended to synthesize brevipolides K and L by esterification of this furanyl alcohol **107** with (*Z*)-4-OMe- and (*Z*)-4-OPMB-cinnamic acid **118** and **119**, respectively. Unexpectedly, olefin isomerization occurred which led to the formation of brevipolides M (**13**) and N (**15**) after treatment with titanium tetrachloride in ≈85% yield each.

### Biological activities

The extracts of *Hyptis brevipes* Poit. have been utilized by humanity across the world in tropical regions ranging from folk medicines [2–3,5–10] to pest management [2–3]. These traditional applications stimulated researchers to conduct biological studies with these plant extracts. A variety of bioactivities were then evidenced, such as antifungal, antibacterial [4,9] insecticidal [7], and radical scavenging [2]. Interestingly, the extracts also exhibited promising DNA intercalating agents [9] and antitumor activity [2]. The latter became more obvious when the chemical constituents of *Hyptis brevipes* Poit., the brevipolides A–F (**1**–**6**), were isolated for the first time by Kinghorn and co-workers in 2009 [4]. These compounds demonstrated cytotoxicity against various human cancer cells. Additionally, the research group led by Pereda-Miranda later isolated more members of brevipolide (**1**–**15**) from the same plant during 2013–2017 and further confirmed the cytotoxic activity of these natural products [1,12].

The biological activity data of brevipolides A–O (**1**–**15**) and their analogs from literature has been summarized in Tables 1–3. Table 1 presents the cytotoxicity data of compounds **1**–**15** and their analogs, if existing, against a range of cancer cell lines.

**Table 1:** Cytotoxicity data of brevipolides and their derivatives against selected cancer cell lines.^a^

entry	compounds	ED_50_ or IC_50_ (μM)								reference

		Lu1	HT-29	HCT-15	MCF-7	Hep-2	HeLa	PC-3	KB	

1	**1**	NS	*5.8* ^b^	NT	>10^b^	NT	NT	NT	NT	[4]
2	**2**	NS	6.1^b^	NT	6.1^b^	NT	NT	NT	NT	[4]
3	**3**	NS	NS	NT	>10^b^	NT	NT	NT	NT	[4]
4	**4**	NS	NS	NT	>10^b^	NT	NT	NT	NT	[4]
5	**5**	NS	NS	NT	>10^b^	NT	NT	NT	NT	[4]
6	**6**	NS	7.5^b^	NT	6.7^b^	NT	NT	NT	NT	[4]
7	**7**	NS	NS	21.7^c^	*3.6*–*5.1*^b^	*8.8* ^c^	*0.18* ^c^	13.2^c^	*0.8* ^c^	[4,11–12]
8	**8**	NT	NT	17.0^c^	5.2^b^	20.7^c^	14.5^c^	13.5^c^	5.0^c^	[11–12]
9	**9**	NT	NT	*14.0* ^c^	17.7^c^	15.7^c^	11.0^c^	*11.7* ^c^	4.7^c^	[11–12]
10	**10**	NT	NT	25.7^c^	*8.4* ^c^	14.6^c^	5.5^c^	13.9^c^	2.9^c^	[12]
11	**11**	NT	NT	>24.8^c^	>24.8^c^	>24.8^c^	10.4^c^	24.8^c^	23.6^c^	[1]
12	**12**	NT	NT	>25.7^c^	23.9^c^	19.8^c^	8.5^c^	22.7^c^	19.3^c^	[1]
13	**13**	NT	NT	>24.8^c^	>24.8^c^	14.2^c^	14.9^c^	18.6^c^	4.2^c^	[1]
14	**14**	NT	NT	>25.7^c^	>25.7^c^	>25.7^c^	24.4^c^	>25.7^c^	9.3^c^	[1]
15	**15**	NT	NT	>24.7^c^	>24.7^c^	23.2^c^	17.1^c^	>24.7^c^	12.1^c^	[1]
16	*ent*-**8**	NT	NT	NT	NT	NT	NT	19.2^c^	NT	[16]
17	**41**	NT	NT	NT	22.4^c^	NT	NT	NT	NT	[14]
18	**43**	NT	NT	NT	*7.0* ^c^	NT	NT	NT	NT	[14]

^a^Abbreviations: Lu1 = lung carcinoma; HT-29 = colon carcinoma; HCT-15 = colon carcinoma; MCF-7 = breast carcinoma; Hep-2 = laryngeal epidermoid carcinoma; HeLa = cervix carcinoma; PC-3 = prostate carcinoma; KB = nasopharyngeal carcinoma; NS = not significant; NT = not tested. ^b^ED_50_. ^c^IC_50_.

A cytotoxicity study against Lu1 human lung cancer cells was conducted for brevipolides A–G (**1**–**7**) by Kinghorn and co-workers but none of the compounds showed significant activity (Table 1, entries 1–7) [4]. These seven compounds were also evaluated against HT-29 human colon carcinoma cells and only three of them were potent (Table 1, entries 1, 2, and 6) with the lowest ED_50_ value of 5.8 μM obtained for brevipolide A (**1**) (Table 1, entry 1). Similar cytotoxicity evaluation was performed for brevipolides G–O (**7**–**15**) against HCT-15 human colon cancer cells (Table 1, entries 7–15), and relatively high ED_50_ values were obtained with the lowest number of 14.0 μM observed for brevipolide I (**9**) (Table 1 entry 9) [1,12]. Moving forward, cytotoxicity examination against MCF-7 human breast carcinoma cells was done for all the brevipolide members (Table 1, entries 1–15) [1,4,12]. Brevipolides A–H (**1**–**8**) were evaluated in vivo (Table 1, entries 1–8), and the highest cytotoxicity against MCF-7 was obtained for brevipolide G (**7**) with ED_50_ values ranging between 3.6–5.1 μM (Table 1, entry 7). The remaining members of brevipolides were evaluated in vitro against MCF-7 cells (Table 1, entries 9–15), and the best result was obtained for brevipolide J (**10**) with an IC_50_ value of 8.4 μM (Table 1, entry 10). Interestingly, brevipolide analogue **43**, which is the reduced form of 6’-*epi*-brevipolide H, showed even better in vitro cytotoxicity with an IC_50_ value of as low as 7.0 μM (Table 1, entry 18) [14]. Also an evaluation on Hep-2 human laryngeal epidermoid cancer cells was conducted for brevipolides G–O (**7**–**15**) (Table 1, entries 7–15), and the lowest IC_50_ value (8.8 μM) was observed for brevipolide G (**7**) (Table 1, entry 7) [1,12]. These nine compounds were also evaluated against HeLa, PC-3, and KB human cervix, prostate, and nasopharyngeal cancer cells, respectively, with the lowest IC_50_ values successively obtained for brevipolides G (**7**), I (**9**) and G (**7**) (0.18, 11.7, and 0.8 μM, respectively) (Table 1, entries 7, 9, and 7) [1,12]. The non-natural brevipolide H (*ent*-**8**) was also evaluated against PC-3 human prostate carcinoma cells but gave no better cytotoxicity results as compared to the natural version (Table 1, entry 16 vs entry 8) [16,19].

Table 2 summarizes the results of other important biological evaluations for brevipolides A–I (**1**–**9**). The mitochondrial transmembrane potential assay was investigated for brevipolides A–G (**1**–**7**) (Table 2, entries 1–7). A very low ED_50_ value of 8.5 nM was obtained for brevipolide C (**3**) (Table 2, entry 3) [4], making it a very potent lead compound for anticancer candidates related to mitochondrial dysfunction effect. A reduction in the mitochondrial membrane potential (MMP) could also indicate apoptosis [20]. These seven compounds, **1**–**7**, were also evaluated for enzyme-based ELISA NF-κB and proteasome inhibition assays (Table 2, entries 1–7), but only brevipolide G (**7**) and brevipolide C (**3**) showed significant activities with ED_50_ values of 15.3 and 38.0 μM, respectively (Table 2, entries 7 and 3) [4]. Lastly, brevipolides G–I (**7**–**9**) were found to inhibit the CCR5 receptor signaling as measured by a calcium mobilization assay with IC_50_ values of 15.5, 13.7, and 18.0 μM, respectively, which make them potential agents for treating HIV disease (Table 2, entries 7–9) [11].

**Table 2:** Summary of other biological evaluations for brevipolides A–I (**1**–**9**) found in the literature.^a^

entry	compound	ED_50_ or IC_50_ (μM)				reference

		mitochondrial transmembrane potential assay	enzyme-based ELISA NF-κB assay	proteasome inhibition assay	chemokine CCR5 receptor	

1	**1**	NS	>50^b^	NS	NT	[4]
2	**2**	NS	>50^b^	NS	NT	[4]
3	**3**	*0.0085* ^b^	>50^b^	*38.0* ^b^	NT	[4]
4	**4**	NS	>50^b^	44.5^b^	NT	[4]
5	**5**	NS	>50^b^	NS	NT	[4]
6	**6**	NS	>50^b^	NS	NT	[4]
7	**7**	0.075^b^	*15.3* ^b^	NS	15.5^c^	[4,11]
8	**8**	NT	NT	NT	*13.7* ^c^	[11]
9	**9**	NT	NT	NT	18.0^c^	[11]

^a^Abbreviations: NS = not significant; NT = not tested. ^b^ED_50_. ^c^IC_50_.

Giménez and co-workers, in 2019, reported the antiparasitic study of brevipolides C, G, H, and J (**3**, **7**, **8**, and **10**) and the IC_50_ (mM) data obtained are summarized in Table 3 [5]. These compounds demonstrated varying activity levels against multiple *Leishmania* strains, *Trypanosoma cruzy*, *Plasmodium falciparum*, and *Giardia lamblia* (Table 3, entries 1–8). Except for *G. lamblia*, brevipolide H (**8**) showed the best antiparasitic activity against all the tested human intestine parasites with IC_50_ values of 12.5–50.4 mM. Thus, this compound has great potential as a lead structure in parasite research. A cytotoxicity study of these four compounds against HeLa human cervix cancer cells was also performed, and the results showed values spanning between 47.2–70.8 mM (Table 3, entry 9). However, these results were considerably much lower than those obtained by the Kinghorn [4] and Pereda-Miranda [12] research groups, which lied in a micromolar order (see Table 1, entries 3, 7, 8, and 10). This prominent difference might arise from the differing methods used.

**Table 3:** IC_50_ (mM) values of **3**, **7**, **8**, and **10** (brevipolides C, G, H, and J) against *protozoa* parasites and HeLa cells.^a^

entry	*protozoa* parasites and cancer cells	compounds			

		**3**	**7**	**8**	**10**

1	Lma	202.0 ± 8.0	62.1 ± 23.3	*18.7 ± 6.7*	24.0 ± 7.2
2	Lae	>248.5	121.6 ± 16.6	*17.5 ± 3.5*	41.5 ± 1.0
3	M2904	182.4 ± 67.1	155.5 ± 32.1	*12.5 ± 1.5*	120.3 ± 36.3
4	LbG	167.2 ± 59.1	>258.8	*32.5 ± 15.0*	146.5 ± 35.1
5	Llan	>248.5	155.5 ± 18.1	*23.5 ± 1.0*	64.8 ± 26.4
6	*T.c*	>248.5	>258.8	*50.4 ± 8.7*	149.8 ± 17.3
7	*P.f*	54.4 ± 18.6	56.9 ± 9.8	34.0 ± 16.0	*34.6 ± 13.2*
8	*G.l*	>248.5	111.3 ± 36.2	76.7 ± 10.0	*43.2 ± 0.7*
9	HeLa	*47.2 ± 7.7*	82.8 ± 4.7	94.9 ± 4.0	70.8 ± 9.4

^a^Abbreviations: Lma: *Leishmania amazonensis*; Lae: *L. aethiopica*; M2904: *L. braziliensis*; LbG: *L. braziliensis native*; Llan: *L. lainsoni, native*; *T.c*: *Trypanosoma cruzi*; *P.f*: *Plasmodium falciparum*; *G.l*: *Giardia lamblia*; HeLa: human cervix carcinoma.

In the same year, Borges and co-workers performed an ovicidal activity evaluation against *Haemonchus placei* eggs using the extract of the *Hyptis brevipes* plant [21]. In tropical areas, *H. placei* is particularly harmful to cattle, causing hypoproteinemia, anemia, and anorexia in the host animals [22]. The extract showed hatchability inhibition with ED_50_ and EC_50_ values of 3.34 and 5.71 mg/mL, respectively. A complete inhibition was achieved at the concentration of 25 mg/mL of the *H. brevipes* extract. Analysis of data combination from ovicidal activity, mass spectrometry, and metabolomics using HPLC-diode array detector-MS (HPLC-DAD-MS), partial least squares regression analysis (PLS-DA), and a correlation map (univariate correlation analyses) enabled the prediction of compounds that have a positive correlation with biological activity. This analysis attributed two detected [M − H]^−^ signals to dihydrobrevipolides C and F. Nevertheless, further structural identification was not performed.

## Conclusion

The characteristic structural features of 5,6-dihydro-α-pyrone derivatives isolated from *Hyptis brevipes* Poit., i.e., the brevipolides A–O, parallel with their appealing biological activity, in addition to the fact that they were produced in a small quantity from nature, make these natural products highly relevant when considering total synthesis. Since the first attempt to prepare brevipolide H by Kumaraswamy in 2014, several model, formal, and total syntheses of brevipolides have been performed, adding more understanding to the chemical and biological aspects of these compounds. In particular, in this review, the main strategies for the synthesis of brevipolides involve: 1) olefin metathesis; 2) asymmetric dihydroxylation and epoxidation; 3) asymmetric hydrogenation; 4) Horner–Wadsworth–Emmons olefination; and 5) cyclopropanation, which are summarized in Table 4, including the overall yields and the number of steps required.

**Table 4:** Summary of the syntheses described in this review.

synthesis	products	key transformations	overall yield	number of steps

Kumaraswamy [14], 2014	reduced 6’-*epi*-brevipolide H (**43**)	C4-C5: ring-closing metathesis C5-C6: vinyl Grignard addition C1’-OH: VO-catalyzed epoxidation C6’-OH: catalytic asymmetric transfer hydrogenation cyclopropane ring: Furukawa’s modified Simmons–Smith	2.5% from **20**	23
Hou [16], 2014	*ent*-brevipolide H (*ent*-**8**)	C4-C5: ring-closing metathesis C2’-C4’: cross metathesis C6’-OH: Sharpless asymmetric dihydroxylation cyclopropane ring: Michael-initiated ring closure	10% from *ent*-**49**	11
Mohapatra [13], 2015	C1-C12 fragment of reduced 6’-*epi*-brevipolide H (**77**)	C3=C4: ring-closing metathesis C5-C6: Brown asymmetric allylation C1’-OH: anti-selective reduction C6’-OH: Jørgensen epoxidation cyclopropane ring: Furukawa’s modified Simmons–Smith	12.5% from **62**	18
Hou [15], 2016	brevipolide H (**8**)	C3=C4: ring-closing metathesis C5-C6: vinyl Grignard addition C6-OH: Sharpless epoxidation C2’-C4’: cross metathesis C6’-OH: Sharpless epoxidation cyclopropane ring: Furukawa’s modified Simmons–Smith	3.5% from **85**	15
Sabitha [17], 2017	brevipolide M (**13**)	C3=C4: ring-closing metathesis C5-C6: Brown asymmetric allylation C3’-C4’: Horner–Wadsworth–Emmons olefination C5’-C6’: Horner–Wadsworth–Emmons olefination C5’-OH: Sharpless epoxidation C6’-OH: Sharpless epoxidation, Mitsunobu inversion furan ring: acid-catalyzed cyclization	5.8% from **97**	17
Sabitha [18], 2018	brevipolide M (**13**) and N (**14**)	C3=C4: Lindlar reduction C5-C6: alkyne addition C3’-C4’: Horner–Wadsworth–Emmons olefination C5’-OH: Noyori reduction furan ring: acid-catalyzed cyclization	20.2% from **111**	11

This work is expected to provide useful information for researchers to design new synthetic methodologies for reproducing these natural products and their analogues as well as to develop new pharmaceuticals from them.
